# An Evaluation of Levalbuterol HFA in the Prevention of Exercise-Induced Bronchospasm

**DOI:** 10.1080/02770900701595667

**Published:** 2007-11-08

**Authors:** D.S. Pearlman, William Rees, Kendyl Schaefer, Holly Huang, William T. Andrews

**Affiliations:** 1 Colorado Allergy and Asthma Centers, P.C., Denver, Colorado, U.S.A.; 2 PI-Coor Clinical Research LLC, Burke, Virginia, U.S.A.; 3 Sepracor Inc., Marlborough, Massachusetts, U.S.A.

**Keywords:** levalbuterol HFA, exercise-induced bronchospasm, β_2_-agonist, efficacy, metered dose inhaler (MDI)

## Abstract

**Background:**

Exercise-induced bronchospasm (EIB) affects up to 90% of all patients with asthma.

**Objective:**

This study evaluated the ability of levalbuterol hydrofluoroalkane (HFA) 90 μg (two actuations of 45 μg) administered via metered dose inhaler (MDI) to protect against EIB in mild-to-moderate asthmatics.

**Methods:**

This was a randomized, double-blind, placebo-controlled, two-way cross-over study. Patients with asthma (*n* = 15) were ≥18 years, had a ≥6-month history of EIB, ≥70% baseline predicted forced expiratory volume in 1 second (FEV_1_), and a 20% to 50% decrease in FEV_1_ after treadmill exercise challenge using single-blind placebo MDI. Levalbuterol or placebo was self-administered 30 minutes before exercise. Treatment sequences were separated by a 3-to 7-day washout period. Spirometry was performed predose, 20 minutes postdose/pre-exercise, and 5, 10, 15, 30, and 60 minutes post-exercise. The primary endpoint was the maximum percent decrease in FEV_1_ from baseline (postdose/pre-exercise). The percentage of protected (≤20% decrease in post-exercise FEV_1_) patients was also assessed.

**Results:**

Levalbuterol had significantly smaller maximum percent post-exercise decrease in FEV_1_ compared with placebo (LS mean ± SE; −4.8% ± 2.8% versus −22.5% ± 2.8%, respectively). For levalbuterol, 14/15 (93.3%) patients had <20% decrease in post-exercise FEV_1_ compared with 8/15 (53.3%) for placebo (*p* = 0.0143). Treatment was well tolerated.

**Conclusion:**

Levalbuterol HFA MDI (90 μg) administered 30 minutes before exercise was significantly more effective than placebo in protecting against EIB after a single exercise challenge and was well tolerated.

**Clinical Implications:**

Levalbuterol HFA MDI when administered before exercise was effective in protecting adults with asthma from EIB.

## Introduction

Exercise-induced bronchospasm (EIB) is a common condition occurring in up to 90% of patients with asthma and in up to 10% of patients that are not known to be asthmatic (1, 2). EIB is an acute transient airway narrowing that occurs sometimes during or very often after exercise. It is thought to be initiated by abrupt changes in airway temperature and humidity during exercise (1). It is particularly common in children and young adults (3), most likely due to their higher physical activity. EIB often results in affected persons withdrawing from physical activity (4–6). The current asthma treatment guidelines recommend pre-treating EIB by administering short-acting β_2_-agonists shortly before exercise, which can protect over 80% of the patients (7).

Two commonly prescribed short-acting β_2_-agonists used for treating asthma are racemic albuterol and levalbuterol. Racemic albuterol is a 1:1 mixture of (R)- and (S)-albuterol. The (R)-albuterol enantiomer is responsible for the bronchoprotective and bronchodilatory properties of racemic albuterol (8, 9). Levalbuterol is the (R)-albuterol enantiomer and is available as a hydrofluoroalkane (HFA) metered dose inhaler (MDI) or as a solution for nebulization. Pre-clinical studies suggest that (S)-albuterol may be pro-inflammatory and pro-constrictive and may attenuate the beneficial effects of (R)-albuterol (10). The clinical relevance of these data for the treatment of asthma is not clear.

Several studies have demonstrated the ability of racemic albuterol MDI to protect against EIB (11–15). Although the FDA has not approved levalbuterol HFA MDI to treat EIB, we were interested in evaluating the protective effect of levalbuterol in asthmatics. This is the first reported study to investigate and describe that effect.

## Methods

### Study Design

This was a randomized, double-blind, placebo-controlled, multicenter, two-way cross-over study designed to evaluate the efficacy of levalbuterol HFA MDI (90 μg; two actuations of 45 μg each) versus placebo in subjects with asthma 18 years of age and older with EIB. The study was conducted according to the principles established by the Declaration of Helsinki. The appropriate Institutional Review Boards approved the protocol, and written informed consent was obtained from the patients.

### Study Patients

Eligible patients were required to be ≥18 years of age with stable asthma; have a forced expiratory volume in 1 second (FEV_1_) ≥70% of predicted, and have been using β_2_-adrenergic agonists, anti-asthma, anti-inflammatory medication, or over-the-counter asthma medication for at least 6 months. Patients were required to have a documented history of EIB for a minimum of 6 months and a decrease in FEV_1_ of at least 20% but no more than 50% after a dose of single blind placebo MDI (HFA-134a propellant containing ethanol and oleic acid; two actuations) and a baseline exercise challenge. One patient with a maximum percent decrease in FEV_1_ of 19% was included in the study. Patients were ineligible if they were diagnosed with life-threatening asthma (intubation, hypercapnia, respiratory arrest, hypoxic seizures) within 12 months before the first study visit. They were also not eligible if they had been hospitalized with asthma within 4 weeks of the first visit or had a significant upper or lower respiratory infection 3 weeks before the first study visit. Patients were excluded if they had a documented history of bronchopulmonary aspergillosis or allergic alveolitis or had greater than 10-pack-year history of smoking or tobacco product use within 6 months of the initial visit.

Medications that were disallowed during the entire study period included parenteral or oral corticosteroids, all adrenergic bronchodilators (except the study medication), nonprescription asthma medication, and ipratropium bromide. Leukotriene inhibitors and low to moderate doses (≤660 μg fluticasone/day or ≤800 μg beclomethasone/day) of inhaled corticosteroids were allowed provided the patient was maintained on a stable dose 30 days before visit 1. Inhaled corticosteroids were withheld for 10 hours before each study visit. Antihistamines were also allowed but had to be withheld for 48 hours before each study visit.

### Study Protocol

The study consisted of three visits, each separated by 3 to 7 days. At the screening visit (visit 1), all baseline values were obtained. This included an exercise challenge to confirm the diagnosis of EIB per protocol limits. At visit 2, patients were randomly assigned to one of two treatments (levalbuterol HFA MDI [90 μg, two actuations of 45 μg each] or placebo [HFA-134a propellant containing ethanol and oleic acid; two actuations]). Randomization occurred separately within each site using permutated blocks of four. At visit 3 patients were crossed-over to the alternate treatment. At visit 1, subjects received an open-label levalbuterol HFA MDI to be used as rescue medication during the study. Rescue medication was used as-needed except on the day of a clinic visit when it was withheld for at least 8 hours before the visit. Patients also received medical event calendars for monitoring adverse events and concomitant medication.

A standard exercise challenge on a treadmill was performed at 30 minutes postdose (16). During the exercise challenge, the gradient and speed of the treadmill were adjusted at 1-minute intervals to achieve at least 85% of the subject's maximum predicted heart rate (calculated as 220 minus the patient's age). At the qualifying exercise challenge (visit 1), the settings for the treadmill rate and incline at each time interval were recorded. These settings were used for the initial settings at each subsequent exercise challenge with minor adjustments to maintain the subject's target maximum heart rate (85% of maximum). The exercise challenge was concluded when patients maintained the target heart rate for a minimum of 4 minutes. The subject could also stop the test at any time. To control for temperature and humidity of the inspired air during the exercise challenge, subjects inspired compressed dry air at room temperature through a Hans Rudolph Nasal and Mouth Breathing Face Mask with a T-shape configuration and a Two-Way Non-Rebreathing Valve (Hans Rudolf, Inc., Kansas City, Missouri, USA). The temperature of the room was maintained between 68°F to 77°F.

Before the exercise challenge, patients self-administered (two actuations) single-blind placebo MDI (visit 1) or randomized double-blind medication (visits 2 and 3). The MDI was primed (four test sprays) before use by study personnel before dispensing and the subject was instructed on its proper use. Study personnel monitored proper administration of the drug. Spirometry was performed predose/pre-exercise, 20 minutes postdose/pre-exercise (e.g., 10 minutes before exercise), and at 5, 10, 15, 30, and 60 minutes post-exercise (±1 minute). After 60 minutes, spirometry measurements continued every 15 minutes until the subject returned to within 5% of the predose FEV_1_. If the subject's FEV_1_ values did not return to the pre-exercise level within 4 hours, the visit was concluded at the Investigator's discretion. A final FEV_1_ was measured before discharge.

Heart rate was monitored each minute during exercise. Vital signs were measured before and immediately after exercise challenge, before the 30- and 60-minute spirometry measurements, and at discharge. Adverse events were collected from the time of informed consent to the end of study.

The primary endpoint was the maximum percent decrease in FEV_1_ from visit postdose/pre-challenge FEV_1_. Secondary endpoints included the time to FEV_1_ recovery after exercise challenge (defined as the amount of time post-challenge needed for FEV_1_ to return to within 5% of the postdose/pre-challenge value), the mean percent change in FEV_1_ from visit predose to postdose/pre-challenge to post exercise, and the number of protected/unprotected patients. Protected patients were defined as those who had less than a 20% decrease in FEV_1_. These values were based on guidelines (USDHHS Guidance for Industry) that consider patients with a greater than 20% decrease in FEV_1_ after exercise challenge as not being protected from EIB (17). Analyses of protection at other levels (15% and 10%) considered by other investigators to be protective (16, 18) were also analyzed. Safety endpoints included adverse events and vital signs.

### Statistical Analysis

Assuming a within-subject standard deviation of 12 percentage points and based on a two-tailed test with α = 0.05, a sample size of 14 subjects was determined to provide at least 80% power to detect a difference of 10% points in the maximum percent decrease in FEV_1_ from visit postdose/pre-challenge between the two treatment groups. The intent-to-treat (ITT) population consisted of all randomized subjects who received at least one dose of study medication. All efficacy and safety analyses were performed on the ITT population.

The primary endpoint, the maximum percent FEV_1_ decrease from visit postdose/pre-challenge, was analyzed using a mixed model analysis of variance with the maximum percent FEV_1_ decrease as the dependent variable, sequence, treatment, period, and postdose/pre-challenge FEV_1_ as fixed effects, and patient as a random effect (19, 20). The time to FEV_1_ recovery and the number of subjects with less than 10%, 10% to 20%, and more than 20% decrease in FEV_1_ were summarized by treatment. Other secondary endpoints were analyzed using the same mixed model as the primary endpoint. Percent change from pre-dose and visit predose/post-challenge was summarized for FEV_1_ over time by treatment. The frequency of rescue medication and percent of subjects with adverse events were summarized by treatment.

## Results

Of 23 patients screened for the study, 15 met the protocol criteria and were randomized to treatment and all 15 completed the study. The mean age was 30.5 years and the majority (*n* = 13) were male (Table 1). Patients had mild-moderate asthma (mean FEV_1_ 86% of predicted), and all had documented EIB. At study entry, 5/15 (33%) of patients were on inhaled corticosteroids.

**Table 1:** Demographics and baseline characteristics.

	ITT population (*n* = 15)
Age, yr, mean (SD)	30.5 (9.0)
Male, *n* (%)	13 (86.7)
Race, *n* (%)	
Caucasian	15 (100)
FEV_1_, (L), mean (SD)	3.8 (0.6)
% Predicted FEV_1_, mean (SD)	86.5 (9.2)
Maximum % decrease in FEV_1_ postdose/pre-challenge at screening, mean (SD)	−26.5 (7.0)
Patients with allergic rhinitis, *n* (%)	15 (100)
Concomitant medication during the double blind period, *n* (%)	
Montelukast sodium	1 (6.7)
Inhaled corticosteroids	5 (33.3)
Antihistamines	2 (13.3)

### Spirometry Measurements

**Figure 1:**
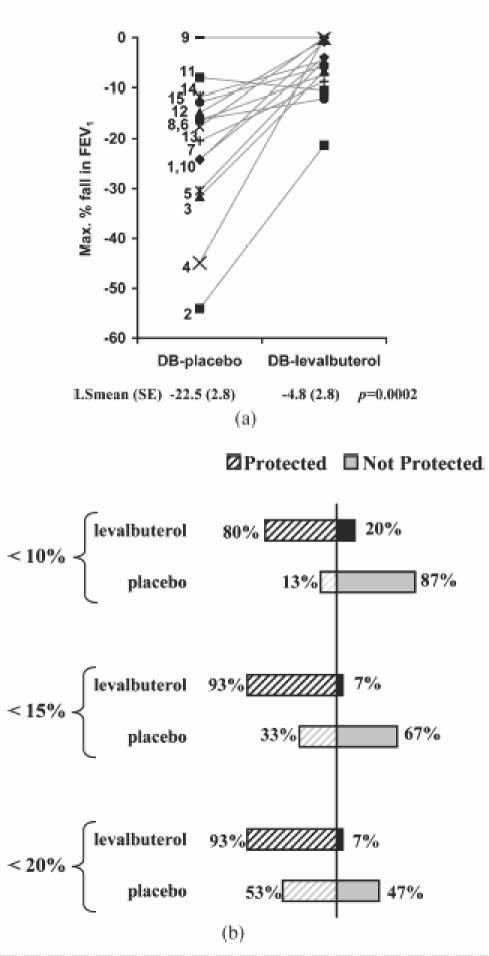
(A) Maximum percent decrease in FEV_1_ for each patient from postdose/pre-challenge after treatment with placebo (DB-placebo) or levalbuterol (DB-levalbuterol) during the double-blind period for each patient. Patient numbers (see Table 2) are indicated and the solid grey lines are to aid following individual patient values. LS-mean (Least square mean) is the adjusted means for the effects in the model (see methods). (B) Percent of patients protected at 10%, 15%, and 20% is indicated.

**Figure 2:**
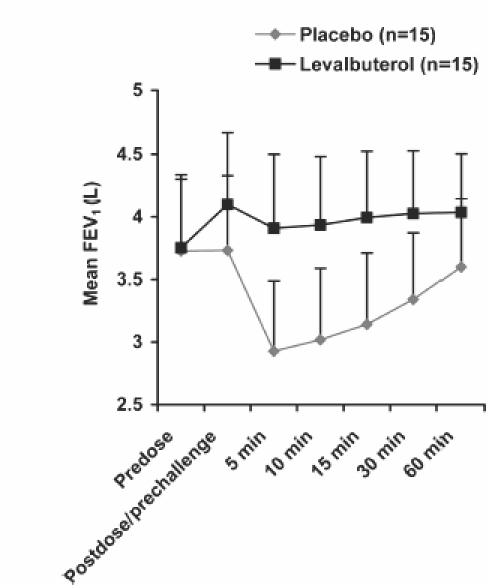
The mean FEV_1_ predose, postdose/pre-exercise challenge, and at each time point post-exercise after treatment with levalbuterol or placebo. The standard deviation for each point is indicated.

The maximum percent decrease in FEV_1_ from visit postdose/pre-challenge (primary endpoint) was significantly less in patients treated with levalbuterol compared with placebo (LS mean ± SE: −4.8% ± 2.8% and −22.5% ± 2.8%, respectively; *p* = 0.0002) (Figure 1A). The mean decrease in FEV_1_ from postdose/pre-challenge at each time point after exercise was also less for levalbuterol than for placebo (Figure 2). More patients were protected against EIB with levalbuterol than placebo. Only 1 of 15 (6.7%) patients (patient 2; see Table 2) treated with levalbuterol had a more than 20% decrease in FEV_1_ compared with 7 of 15 (46.7%) patients for placebo (Figure 1B). In contrast, 12 of 15 (80%) patients after levalbuterol use had less than 10% decrease in FEV_1_ compared with 2 of 15 (13.3%) for placebo (Figure 1B). The mean time for FEV_1_ recovery was faster with levalbuterol (15.0 minutes) compared with placebo (46.4 minutes).

**Table 2:** Maximum percent decrease in FEV_1_ after exercise from two different baselines: postdose/pre-challenge and predose.*

	Visit postdose/pre-challenge at baseline			Visit pre-dose at baseline		
		
Patients	Screening visit (placebo)	DB-placebo	DB-levalbuterol	Screening visit (placebo)	DB-placebo	DB-levalbuterol
1	−42.6	−24.3	−0.9	−41.7	−25.8	0.0
2	−40.8	−54.1	−21.3	−40.8	−55.7	−1.5
3	−31.0	−31.6	−6.8	−31.2	−33.0	0.0
4	−30.0	−44.9	−0.2	−28.1	−42.8	0.0
5	−27.4	−30.4	−4.5	−27.8	−29.5	0.0
6	−26.4	−16.2	−12.0	−27.5	−18.3	−4.0
7	−25.6	−20.4	−8.8	−26.5	−20.8	0.0
8	−23.4	−16.3	−7.4	−21.0	−17.1	−5.7
9	−23.2	0.0	0.0	−24.6	0.0	0.0
10	−22.9	−24.1	−4.0	−21.5	−20.0	0.0
11	−21.2	−8.0	−10.6	−22.5	−7.3	−5.3
12	−21.3	−14.8	−0.5	−20.3	−15.5	0.0
13	−20.5	−17.6	0.0	−16.9	−18.3	0.0
14	−22.4	−11.7	−4.6	−20.7	−8.9	−0.6
15	−19.0	−12.7	−5.8	−19.4	−13.4	−2.5
Mean (SD)	−26.5 (7.0)	−21.8 (14.0)	−5.8 (5.8)	−26.0 (7.3)	−21.8 (14.2)	−1.3 (2.1)

* DB-placebo and DB-levalbuterol indicates the placebo or levalbuterol treatment, respectively, during the double-blind period of the study.

Levalbuterol was associated with significantly greater bronchodilation before exercise compared with placebo. LS mean (SE) percent change in FEV_1_ from predose to postdose/pre-challenge was 9.4 ± 1.1 for levalbuterol compared with 0.3 ± 1.1 for placebo (*p* < 0.0001). After exercise following levalbuterol treatment, the maximum percent decrease in FEV_1_ values for the majority (9 of 15) of individual patients did not fall below predose levels (Table 2). Moreover, the mean FEV_1_ did not fall below predose levels (Figure 2). In contrast after exercise following placebo treatment, the maximum percent decrease in FEV_1_ values for the majority (14 of 15) of patients fell below predose values (Table 2). Post-exercise, the mean FEV_1_ after placebo treatment dropped below predose values. The LS mean (SE) for the maximum percent decrease in FEV_1_ from predose to postexercise was 9.8 ± 2.2 for levalbuterol and −4.2 ± 2.2 for placebo (*p* = 0.0008).

There was some intrapatient variability in response to exercise challenge. For a number of patients, the changes in FEV_1_ post-exercise for the two placebo treatments (at screening [visit 1] and the double-blind period) were dissimilar (Table 2).

After placebo treatment, three patients required one dose of rescue medication after exercise challenge during the double-blind period of the study, and no patients required rescue medication after levalbuterol treatment. There was no significant difference in heart rate or respiration rates with levalbuterol and placebo treatments after exercise (data not shown).

### Adverse Events

There were four adverse events observed. The reported post-randomization adverse events after levalbuterol treatment were myalgia (*n* = 2) and vomiting (*n* = 1), and after placebo treatment nasopharyngitis (*n* = 1). None were considered by the investigators to be related to treatment. There were no serious adverse events reported during the study.

## Discussion

We examined the ability of levalbuterol HFA 90 μg administered through an MDI to protect adults with asthma from EIB. Levalbuterol HFA taken 30 minutes before treadmill exercise was found to be protective. The mean maximum percent decrease in FEV_1_ from postdose/pre-challenge (primary endpoint) was significantly smaller (*p* < 0.001) for levalbuterol treatment than for placebo. All but 1 of 15 subjects were protected from EIB when using levalbuterol with a maximum decrease in FEV_1_ of less than 20%, a criteria suggested by the USDHHS Guidance for Industry (17). Although the decrease in FEV_1_ was greater than 20% for one patient (patient #2), this patient still had a large degree of protection from EIB after levalbuterol treatment, (maximum percent decrease in FEV_1_ following DB-Placebo was −54.1% compared with −21.3% following DB-Levalbuterol) (Table 2). The fact that levalbuterol improved post-exercise FEV_1_ by 32.8% but the decrease was still greater than 20% may reflect the very low baseline value for this patient (decrease in FEV_1_ after DB-Placebo treatment). All other measurements of pulmonary function, including mean percent change in FEV_1_ at all time points post-exercise and time to FEV_1_ recovery (baseline), were supportive of the effective protection against EIB by levalbuterol. None of the three instances in which rescue medication related to exercise challenges was required occurred when levalbuterol was used pre-challenge.

The degree of protection from EIB can be considered clinically important. After levalbuterol treatment, the majority of patients were protected (12 of 15 had less than a 10% decrease in FEV_1_ and 14 of 15 had less than a 15% decrease after exercise). In contrast after placebo treatment, most patients were not protected against EIB (2 of 15 had less than a 10% decrease in FEV_1_ and 5 of 15 had less than 15% decrease post-exercise). A 10% decrease in FEV_1_ is considered to be indicative of healthy non-asthmatic subjects (18, 21–23), while a 15% or greater fall in FEV_1_ is considered by some investigators and guidelines as a diagnosis of EIB.

Protection by levalbuterol was also associated with a rapid bronchodilating effect achieved pre-exercise challenge. Although postdose/pre-challenge has been a commonly used baseline (17), others have suggested predose to be a more appropriate baseline (24, 25). However, with the exception of one patient, in this study, the degree of bronchodilation after exercise from postdose/pre-challenge was not sufficiently different from predose to alter the conclusions of the study if predose was used for baseline (data not shown). The duration of the protective effect of levalbuterol was not examined in this study.

Asthma is characterized by differences between patients and by intrapatient variability both over short and long periods of time (17). In this study, variability in a patient's response was apparent by the difference in percent change in FEV_1_ between the screening and the double-blind placebo treatments (Table 2). In this context, prescribing physicians as well as patients need to appreciate the potential variability in degree of protection by a single dose of bronchoprotective agents used before exercise. Moreover, consistent (e.g., daily) use of β-adrenergic agents can lead to relative tolerance to the bronchoprotective effect of these agents (15, 26–29), although intervals of use of 72 hours or greater abrogate this effect (28). Long-term anti-inflammatory control of asthma can decrease bronchial reactivity to various asthma triggers such as exercise (30–33).

### Conclusion

This is the first reported evaluation of the ability of levalbuterol HFA MDI used as single dose pre-treatment to protect patients from EIB. It was found that a single dose administered 30 minutes before exercise was effective in protecting against EIB and was well tolerated.

## References

[1] Parsons JP, Mastronarde JG (2005). Exercise-induced bronchoconstriction in athletes. Chest.

[2] Anderson SD, Caillaud C, Brannan JD (2006). Beta2-agonists and exercise-induced asthma. Clin Rev Allergy Immunol.

[3] Clark CJ, Cochrane LM (1999). Physical activity and asthma. Curr Opin Pulm Med.

[4] Meyer R, Kroner-Herwig B, Sporkel H (1990). The effect of exercise and induced expectations on visceral perception in asthmatic patients. J Psychosom Res.

[5] Ergood JS, Epstein LH, Ackerman M, Fireman P (1985). Perception of expiratory flow by asthmatics and non-asthmatics during rest and exercise. Health Psychol.

[6] Panditi S, Silverman M (2003). Perception of exercise induced asthma by children and their parents. Arch Dis Child.

[7] NHLBI (1997). Guidelines for the diagnosis and management of asthma.

[8] Penn RB, Frielle T, McCullough JR, Aberg G, Benovic JL (1996). Comparison of R-, S-, and RS-albuterol interaction with human beta 1-and beta 2adrenergic receptors. Clin Rev Allergy Immunol.

[9] Lotvall J, Palmqvist M, Arvidsson P, Maloney A, Ventresca GP, Ward J (2001). The therapeutic ratio of R-albuterol is comparable with that of RS-albuterol in asthmatic patients. J Allergy Clin Immunol.

[10] Milgrom H (2006). Levosalbutamol in the treatment of asthma. Expert Opin Pharmacother.

[11] Berkowitz R, Schwartz E, Bukstein D, Grunstein M, Chai H (1986). Albuterol protects against exercise-induced asthma longer than metaproterenol sulfate. Pediatrics.

[12] Hawksworth RJ, Sykes AP, Faris M, Mant T, Lee TH (2002). Albuterol HFA is as effective as albuterol CFC in preventing exercise-induced bronchoconstriction. Ann Allergy Asthma Immunol.

[13] Shapiro GS, Yegen U, Xiang J, Kottakis J, Della CG (2002). A randomized, double-blind, single-dose, crossover clinical trial of the onset and duration of protection from exercise-induced bronchoconstriction by formoterol and albuterol. Clin Ther.

[14] Sichletidis L, Daskalopoulou E, Kyriazis G, Kosmidou I, Koupidou S, Pechlivanidis T, Chloros D (1993). Comparative efficacy of salbutamol and salmeterol in exercise-induced asthma. J Int Med Res.

[15] Inman M, O'Byrne P (1996). The effect of regular inhaled albuterol on exercise-induced bronchoconstriction. Am J Respir Crit Care Med.

[16] Crapo RO, Casaburi R, Coates AL, Enright PL, Hankinson JL, Irvin CG, MacIntyre NR, McKay RT, Wanger JS, Anderson SD, Cockcroft DW, Fish JE, Sterk PJ (2000). Guidelines for methacholine and exercise challenge testing-1999. This official statement of the American Thoracic Society was adopted by the ATS Board of Directors, 1999. Am J Respir Crit Care Med.

[17] U.S. Department of Health and Human Services FC (2002). Guidance for Industry;Exercise-induced bronchospasm (EIB)—development of drugs to prevent EIB (draft).

[18] McFadden ER, Gilbert IA (1994). Exercise-induced asthma. N Engl J Med.

[19] Jones B, Kenward M (2003). Design and analysis of cross-over trials (monographs on statistics and applied probability).

[20] Littell RC, Milliken GA, Stroup WW, Wolfinger RD (1996). SAS systems for mixed models.

[21] Sterk PJ, Fabbri LM, Quanjer PH, Cockcroft DW, O'Byrne PM, Anderson SD, Juniper EF, Malo JL (1993). Airway responsiveness. Standardized challenge testing with pharmacological, physical and sensitizing stimuli in adults. Report Working Party Standardization of Lung Function Tests, European Community for Steel and Coal. Official Statement of the European Respiratory Society. Eur Respir J Suppl.

[22] Anderson SD, Lambert S, Brannan JD, Wood RJ, Koskela H, Morton AR, Fitch KD (2001). Laboratory protocol for exercise asthma to evaluate salbutamol given by two devices. Med Sci Sports Exerc.

[23] Task force on recognizing and diagnosing exercise-related asthma, respiratory and allergic disorder in sports (2005). Evidence-based recommendations for the diagnosis of exercise-induced asthma in athletes. Eur Respir Mon.

[24] Senn SJ (1989). The use of baselines in clinical trials of bronchodilators. Stat Med.

[25] Boner AL, Spezia E, Piovesan P, Chiocca E, Maiocchi G (1994). Inhaled formoterol in the prevention of exercise-induced bronchoconstriction in asthmatic children. Am J Respir Crit Care Med.

[26] Johnson M (2006). Molecular mechanisms of beta(2)-adrenergic receptor function, response, and regulation. J Allergy Clin Immunol.

[27] Hancox RJ, Subbarao P, Kamada D, Watson RM, Hargreave FE, Inman MD (2002). Beta2-agonist tolerance and exercise-induced bronchospasm. Am J Respir Crit Care Med.

[28] Haney S, Hancox RJ (2005). Rapid onset of tolerance to beta-agonist bronchodilation. Respir Med.

[29] Hayes MJ, Qing F, Rhodes CG, Rahman SU, Ind PW, Sriskandan S, Jones T, Hughes JM (1996). In vivo quantification of human pulmonary beta-adrenoceptors: effect of beta-agonist therapy. Am J Respir Crit Care Med.

[30] Anderson SD (2004). Single-dose agents in the prevention of exercise-induced asthma: a descriptive review. Treat Respir Med.

[31] Vathenen AS, Knox AJ, Wisniewski A, Tattersfield AE (1991). Effect of inhaled budesonide on bronchial reactivity to histamine, exercise, and eucapnic dry air hyperventilation in patients with asthma. Thorax.

[32] Vidal C, Fernandez-Ovide E, Pineiro J, Nunez R, Gonzalez-Quintela A (2001). Comparison of montelukast versus budesonide in the treatment of exercise-induced bronchoconstriction. Ann Allergy Asthma Immunol.

[33] Pedersen S, Hansen OR (1995). Budesonide treatment of moderate and severe asthma in children: a dose-response study. J Allergy Clin Immunol.

